# Cell Density Impacts Epigenetic Regulation of Cytokine-Induced E-Selectin Gene Expression in Vascular Endothelium

**DOI:** 10.1371/journal.pone.0090502

**Published:** 2014-04-01

**Authors:** Katsuhiko Hamada, Mizuko Osaka, Masayuki Yoshida

**Affiliations:** Department of Life Sciences and Bioethics, Graduate School of Medical and Dental Sciences, Tokyo Medical and Dental University, Tokyo, Japan; Osaka University Graduate School of Medicine, Japan

## Abstract

Growing evidence suggests that the phenotype of endothelial cells during angiogenesis differs from that of quiescent endothelial cells, although little is known regarding the difference in the susceptibility to inflammation between both the conditions. Here, we assessed the inflammatory response in sparse and confluent endothelial cell monolayers. To obtain sparse and confluent monolayers, human umbilical vein endothelial cells were seeded at a density of 7.3×10^3^ cells/cm^2^ and 29.2×10^3^ cells/cm^2^, respectively, followed by culturing for 36 h and stimulation with tumor necrosis factor α. The levels of tumor necrosis factor α-induced E-selectin protein and mRNA expression were higher in the confluent monolayer than in the sparse monolayer. The phosphorylation of c-jun N-terminal kinase and p38 mitogen-activated protein kinase or nuclear factor-κB activation was not involved in this phenomenon. A chromatin immunoprecipitation assay of the E-selectin promoter using an anti-acetyl-histone H3 antibody showed that the E-selectin promoter was highly and specifically acetylated in the confluent monolayer after tumor necrosis factor α activation. Furthermore, chromatin accessibility real-time PCR showed that the chromatin accessibility at the E-selectin promoter was higher in the confluent monolayer than in the sparse monolayer. Our data suggest that the inflammatory response may change during blood vessel maturation via epigenetic mechanisms that affect the accessibility of chromatin.

## Introduction

Vascular endothelial cells (ECs) play a pivotal role in the maintenance of the proper systemic vascular network [1], [2], [3]. The vascular system actively regenerates itself to maintain its integrity and organ function [4]. Compared with mature ECs, those with an angiogenic status have been reported to possess unique characteristics [5]. Vascular ECs also play an important role in acute and chronic inflammation. At the site of inflammation, leukocytes interact with activated ECs via adhesion molecules, resulting in rolling, adhesion, and transmigration [6]. These processes are intimately involved in pathogenesis of inflammatory diseases [7], [8], as well as resolution of inflammation [9], [10]. A proper inflammation cascade is vital for the maintenance of systemic homeostasis; however, it is intriguing to know whether vascular ECs during angiogenesis can induce vascular inflammation similar to mature ECs. To address this question, we conducted a study in which vascular ECs cultured under the sparse condition were compared with those cultured under the confluent condition. It is known that sparse and confluent endothelial cells show different phenotypes including cell growth, apoptosis, and cytoskeleton rearrangement. Moreover, the intracellular signaling mechanisms responsible for these phenotypes have been studied [11], [12], [13], [14], [15]. On the other hand, effect of cell density on endothelial gene regulation is partly understood.

In the present study, we demonstrated that tumor necrosis factor α (TNFα)-induced E-selectin expression levels in ECs was cell density dependent, and this phenomenon may be regulated via epigenetic mechanisms that affect the structure and accessibility of chromatin.

## Materials and Methods

### Cell culture

Human umbilical vein endothelial cells (HUVECs) were purchased from Lonza and cultured in endothelial growth medium-2 (Lonza) at 37°C in a humidified atmosphere containing 5% carbon dioxide. Plastic culture dishes were precoated with 1% gelatin, and HUVECs were used between passages 4 and 5.

To obtain sparse and confluent monolayers, HUVECs were seeded at a density of 7.3×10^3^ cells/cm^2^ and 29.2×10^3^ cells/cm^2^, respectively, and were used 36 h after incubation. The medium was changed at 24 h after seeding cells.

### Antibodies

A monoclonal antibody against E-selectin (clone 7A9) was obtained from the American Type Culture Collection; anti-E-selectin (A-10: sc-137203) and anti-NF-kB p65 (C-20: sc-372) antibodies were obtained from Santa Cruz Biotechnology; anti-phospho-NF-κB p65 Ser 536 (#3031), anti-SAPK/JNK (#9252), anti-p38 MAPK (#9212), anti-phospho-SAPK/JNK (#9251), and anti-phospho-p38 MAPK (#4511) antibodies were obtained from Cell Signaling Technology; anti-lamin A/C (SAB4200236) and anti-actin (A5060) antibodies were obtained from Sigma-Aldrich; an anti-α-tubulin antibody (PM054-7) was obtained from Medical & Biological Laboratories; an anti-acetyl-histone H3 antibody (06-599) was obtained from Millipore; an Alexa Fluor 488-conjugated goat anti-mouse IgG antibody (A11017) was obtained from Life Technologies; horseradish peroxidase (HRP)-linked anti-mouse (NA931V) and anti-rabbit (NA9340V) secondary antibodies were obtained from GE Healthcare.

### Western blot analysis

To obtain total cell lysates, cells were lysed in RIPA buffer after treatment with 1 ng/ml recombinant human TNFα (R&D Systems) for the indicated periods. Cytoplasmic and nuclear lysates were prepared using an NE-PER nuclear and cytoplasmic extraction kit (Thermo Scientific) according to the manufacturer's protocol. Lysates from each condition were separated on 10% or 12.5% sodium dodecyl sulfate (SDS)-polyacrylamide gel and transferred onto Immobilon-P membranes (Millipore). The membranes were blotted using the primary antibodies described above, followed by blotting with the HRP-linked secondary antibodies, and the signals were detected by chemiluminescence using the Pierce Western Blotting Substrate or SuperSignal West Dura Extended Duration Substrate (Thermo Scientific).

### Fluorescence flow cytometry

HUVECs cultured in 10-cm dishes were treated with 1 ng/ml TNFα for 4 h and detached by incubation in HBSS with 5 mM EDTA and 4 mM EGTA for 20 min at 37°C [16]. Cells were incubated with the E-selectin antibody (clone 7A9) for 45 min on ice, followed by incubation with the Alexa Fluor 488-conjugated goat anti-mouse antibody. Data were acquired using the FACSCalibur (Becton-Dickinson) and analyzed using the FlowJo software (Tree Star).

### Quantitative RT-PCR

HUVECs were cultured as described above and treated with 1 ng/ml TNFα for 2 h, followed by actinomycin D (5 µg/ml) (Wako Pure Chemical Industries) for 0, 0.5, and 2 h. RNA was then isolated using an RNeasy Mini Kit (Qiagen) and transcribed into cDNA using the PrimeScript RT Master Mix (Takara Bio). Quantitative PCR analysis was performed using a KAPA SYBR FAST Universal qPCR Kit (Kapa Biosystems) and the Thermal Cycler Dice (Takara Bio). We used the following primer pairs: E-selectin forward, 5′-AAGCCCACATGTGAAGCTGT-3′, and reverse, 5′-CTCCAATAGGGGAATGAGCA-3′; GAPDH forward, 5′-AGTGGATATTGTTGCCATCAAT-3′, and reverse, 5′-CTTGACGGTGCCATGGAATT-3′. The relative expression levels of E-selectin were normalized against those of GAPDH.

### Chromatin immunoprecipitation assay

Chromatin immunoprecipitation (ChIP) was performed as described previously [17], [18], [19]. In brief, approximately 0.75×10^6^ cells were used per condition. HUVECs were treated with 1 ng/ml TNFα for 30 min, followed by incubation with 1% formaldehyde for 10 min. Cells were washed with ice-cold PBS and harvested in ice-cold PBS containing the complete protease inhibitor cocktail (Roche). After rapid centrifugation, the pellets were resuspended in lysis buffer [50 mM Tris (pH 8.0), 10 mM EDTA, 1% SDS, and protease inhibitor cocktail] and incubated for 10 min on ice. Lysates were sonicated on ice to yield 200–400-bp DNA fragments using an ultrasonic homogenizer VP-050 (TAITEC). The sonicated samples were diluted 10-fold using dilution buffer [16.7 mM Tris (pH 8.0), 167 mM NaCl, 1.2 mM EDTA, 0.01% SDS, 1.1% Triton X-100, and protease inhibitor cocktail]. The diluted samples were precleared with 60 µl of Protein A Agarose/Salmon Sperm DNA (Millipore) for 30 min at 4°C, followed by incubation with or without 5 µg of the anti-acetyl-histone H3 antibody overnight at 4°C. Immunocomplexes were precipitated by incubation with 45 µl of Protein A Agarose/Salmon Sperm DNA at 4°C for 60 min. Immunoprecipitates were repeatedly washed, and the complex was eluted in 1% SDS/0.1 M NaHCO_3_. Cross-links were reversed by adding 20 µl of 5 M NaCl to 500 µl of eluate and incubating at 65°C for 6 h. The samples were treated with proteinase K, and DNA was recovered using phenol–chloroform extraction, followed by ethanol precipitation and resuspension in water (30 µl). Quantitative PCR was performed using specific primers for the E-selectin promoter region (−195 to −67 relative to the transcriptional start site); forward, 5′-AGGCATGGACAAAGGTGAAG-3′, and reverse, 5′-GTCCACATCCAGTAAAGAGG-3′; and for the E-selectin extreme 3′ region (+13914 to +13977) [20]; forward, 5′-TGGCAGGCAGAGGAATGG-3′, and reverse, 5′-CCATTTTCCACACCGCTATGA-3. Specific endogenous ChIP enrichments of the E-selectin promoter region were all at least 3-fold compared with the no-antibody ChIP. Data are expressed as %INPUT = 2^−ΔCt^×100 (%), where ΔCt = Ct of sample − Ct of INPUT.

### Chromatin accessibility real-time PCR

Chromatin accessibility real-time PCR (CHART-PCR) was performed as described previously with minor modifications [21], [22]. In brief, HUVECs were treated with 1 ng/ml TNFα for 60 min and scraped into ice-cold PBS. After centrifugation, the cell pellets (approximately 0.6×10^6^ cells/sample) were resuspended in 400 µl of lysis buffer [10 mM Tris-HCl (pH 7.4), 10 mM NaCl, 3 mM MgCl_2_, 0.1% Nonidet P-40, 150 µM spermine, and 500 µM spermidine] and incubated on ice for 5 min. After centrifugation, the pelleted nuclei were washed with 400 µl of wash buffer [10 mM Tris-HCl (pH 7.4), 15 mM NaCl, 60 mM KCl, 150 µM spermine, and 500 µM spermidine]. Subsequently, they were resuspended in 100 µl of digestion buffer (wash buffer containing 1 mM CaCl_2_) and digested with 10 U of micrococcal nuclease (Worthington Biochemical Corporation) at room temperature for 10 min. The reaction was terminated by adding 100 µl of stop buffer (20 mM EDTA, 2 mM EGTA, and 1% SDS), followed by incubation on ice for 10 min. Subsequently, 75 µg of proteinase K was added to the sample before incubating overnight at 50°C. DNA was isolated using phenol–chloroform extraction, followed by ethanol precipitation, and 20 ng of DNA from the micrococcal nuclease-digested sample and the undigested control sample were used for quantitative PCR.

Primers used for the E-selectin promoter region (−153 to −93) included; forward, 5′-TGACATCATTGTAATTTTAAGCATCGT-3′, and reverse, 5′-CCAATGGCATCCAAAAACTTTC-3′. The control primers previously used included; forward, 5′-CAGCTCACCATGGATGATGATATC-3′, and reverse, 5′-TGCCGGAGCCGTTGTC-3′ [20]. Percent protection was calculated as the amount of DNA in the digested sample relative to that in the undigested sample.

### Statistical analyses

All the experiments were performed at least three times, and data are expressed as mean ± SD. Statistical analyses were performed using a paired *t*-test and two-way ANOVA. A *p* value of <0.05 was considered to be statistically significant.

## Results

### TNFα-induced E-selectin expression levels were compromised in HUVECs cultured under the sparse condition

**Figure 1:**
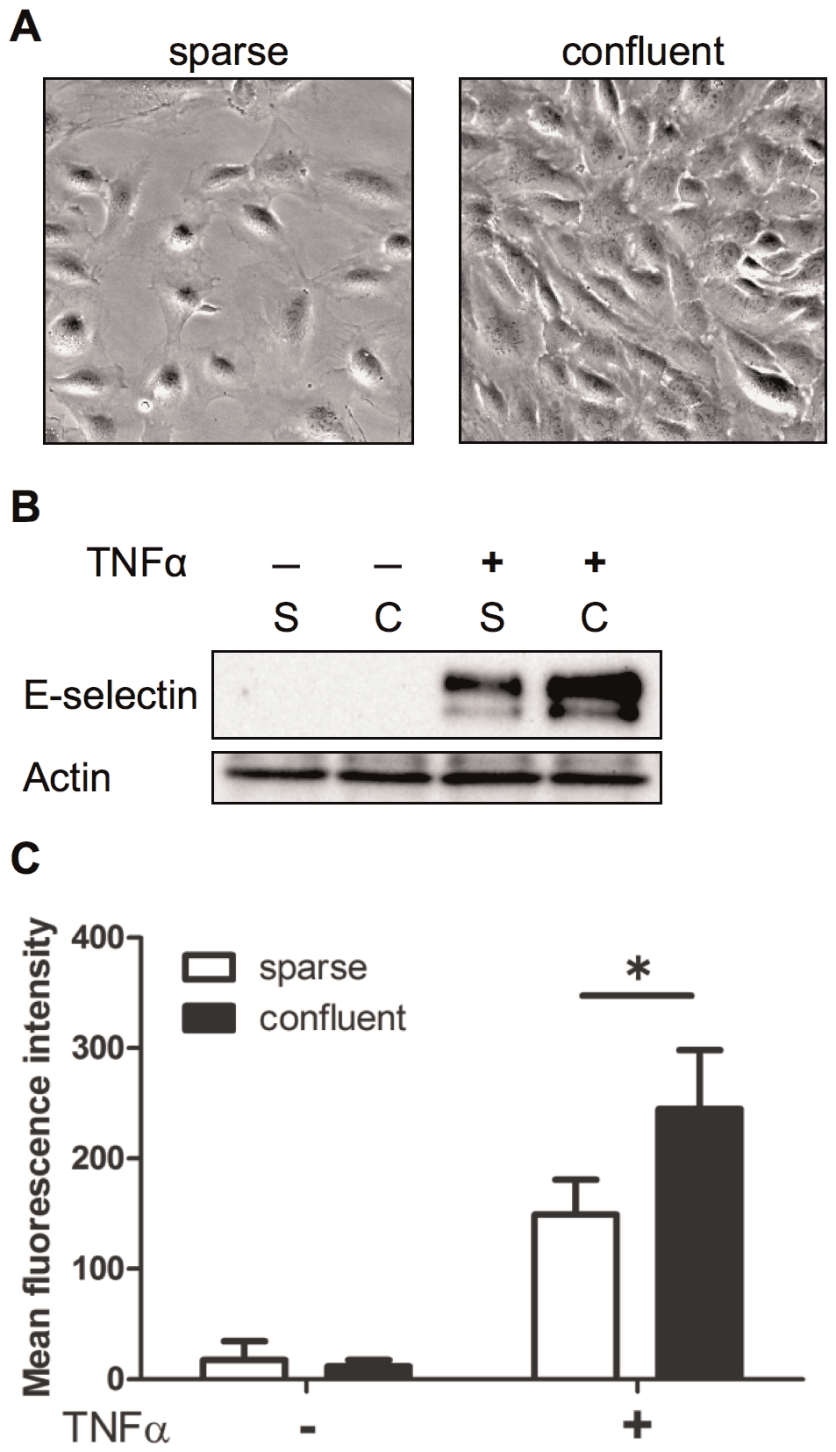
Effect of cell density on E-selectin protein expression levels in HUVECs. (**A**) HUVECs were cultured as described in the Materials and Methods. Photomicrographs were obtained using phase-contrast microscopy (20× magnification). (**B**) E-selectin protein expression levels under the sparse (S) and confluent (C) conditions. HUVECs cultured under both the conditions were treated with (+) or without (−) 1 ng/ml TNFα for 4 h, and total cell extracts were prepared. Equal amounts of protein were separated using SDS-PAGE, and western blot analysis was performed using the anti-E-selectin antibody (Santa Cruz Biotechnology). (**C**) Flow cytometric analysis of E-selectin cell surface expression. HUVECs were stimulated with or without 1 ng/ml TNFα for 4 h, and flow cytometry was performed using the anti-E-selectin antibody (clone 7A9) (*p<0.05). The values are expressed as mean ± SD of three independent experiments.

To investigate the effect of cell density on E-selectin expression levels induced by TNFα, we performed western blot analyses using total extracts obtained from sparse and confluent HUVEC monolayers (Figure 1A). We found that TNFα-induced E-selectin expression levels in the HUVECs was higher in the confluent monolayer than in the sparse monolayer, whereas the expression levels of actin were comparable in HUVECs cultured under these two conditions (Figure 1B). Similar results were obtained using interleukin-1β (data not shown). Flow cytometry analysis of E-selectin showed that the mean fluorescence intensity was also significantly higher in the confluent HUVEC monolayer than in the HUVEC sparse monolayer, thereby indicating that the observed difference in E-selectin expression levels was not due to the difference in the cell number between these two conditions. However, the endothelial response to TNFα may have differed between sparse and confluent monolayers (Figure 1C).

**Figure 2:**
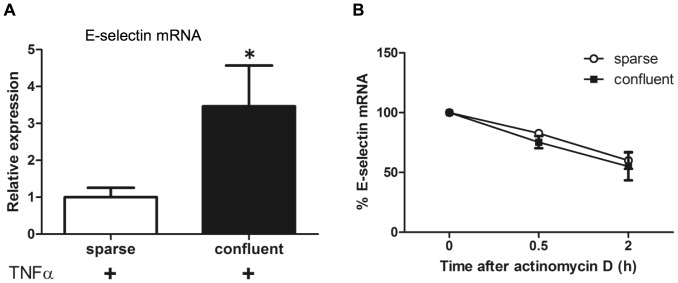
Effect of cell density on E-selectin mRNA expression and mRNA stability in HUVECs. (**A**) Relative E-selectin mRNA levels measured using quantitative RT-PCR. RNA was isolated 2 h after stimulation with 1 ng/ml TNFα and 5 ng cDNA was used in each reaction (*p<0.05 vs sparse). (**B**) E-selectin mRNA stability in TNFα-activated HUVECs. HUVECs were stimulated with 1 ng/ml TNFα for 2 h and treated with actinomycine D (5 µg/ml) for the indicated time periods. RNA was subjected to real-time RT-PCR. For each condition, E-selectin expression levels were normalized against those of GAPDH. The values were expressed proportional to that at baseline (time zero). The values are expressed as mean ± SD of three independent experiments.

Subsequently, we tested whether E-selectin mRNA expression levels is dependent on cell density. Quantitative RT-PCR demonstrated that TNFα-induced E-selectin mRNA expression levels were significantly higher in the confluent HUVEC monolayer than in the HUVEC sparse monolayer (Figure 2A). Monitoring the stability of E-selectin mRNA after treatment with actinomycin D showed that no significant difference was observed in E-selectin mRNA degradation between sparse and confluent monolayers (Figure 2B).

### Mitogen-activated protein kinase- and nuclear factor-κB-dependent pathways were not involved in the cell density-mediated regulation of E-selectin expression levels

**Figure 3:**
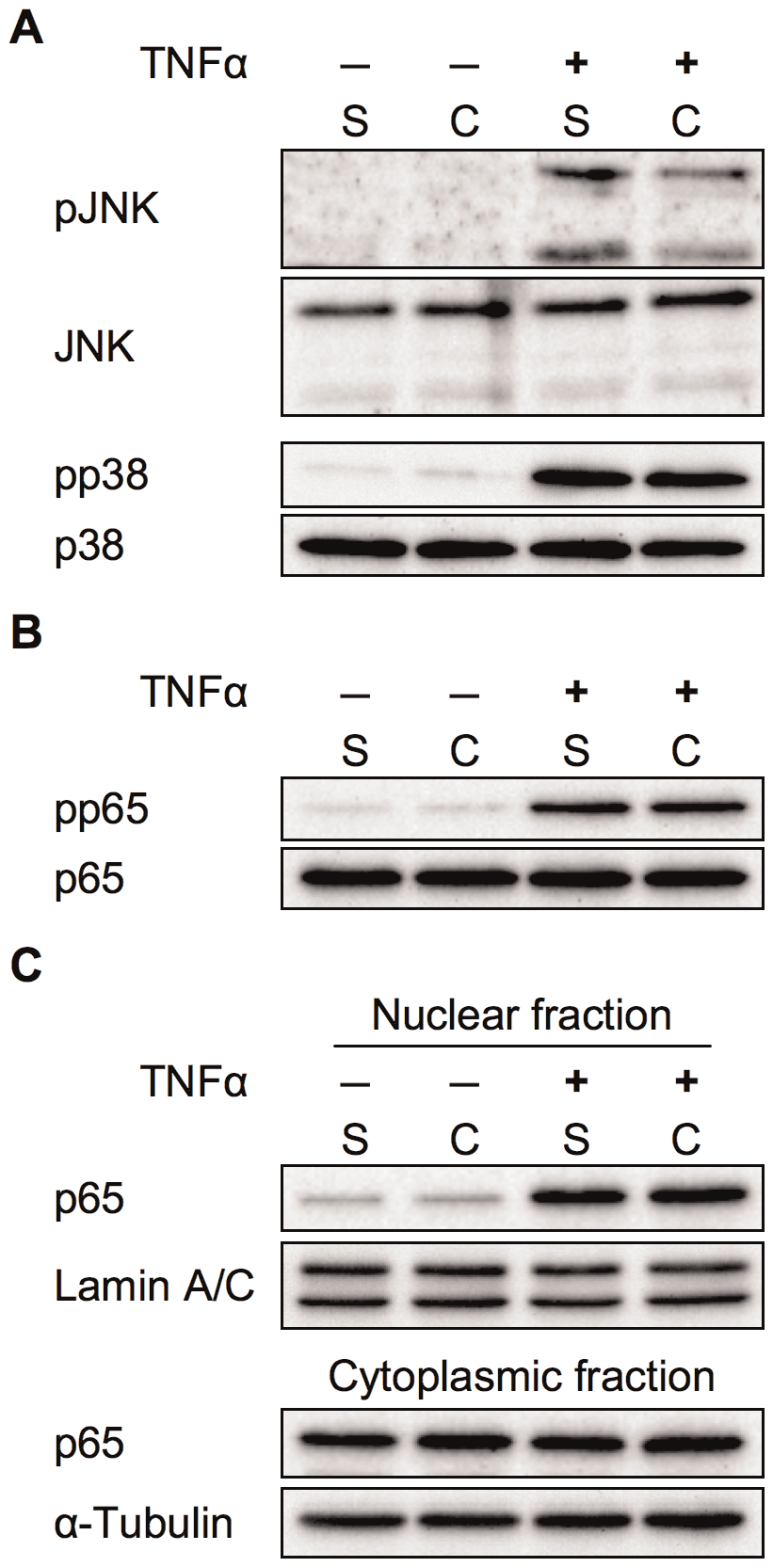
Effect of cell density on TNFα-induced MAPK and NF-kB activation in HUVECs. Western blot of phosphorylated (p) and total JNK, p38 MAPK (**A**), and NF-κB p65 (**B**). HUVECs were stimulated with (+) or without (−) 1 ng/ml TNFα for 15 min. (**C**) p65 was detected using western blot analysis of the nuclear and cytoplasmic fractions prepared from HUVECs treated with or without 1ng/ml TNFα for 30 min. Representative western blots are shown.

We examined the involvement of mitogen-activated protein kinase (MAPK)- and nuclear factor-κB (NF-κB)-dependent pathways, which are the two major transcriptional pathways involved in E-selectin gene expression [23], [24]. Western blot analyses showed that the phosphorylation of c-jun N-terminal kinase (JNK) was decreased under the confluent condition, whereas that of p38 MAPK did not differ between the two conditions (Figure 3A). We also examined the activation of the p65 component of NF-κB; however, no difference was observed in the TNFα-induced phosphorylation of p65 (Figure 3B), as well as its nuclear translocation (Figure 3C).

### Cell density specifically modulated histone acetylation at the E-selectin promoter after TNFα activation

**Figure 4:**
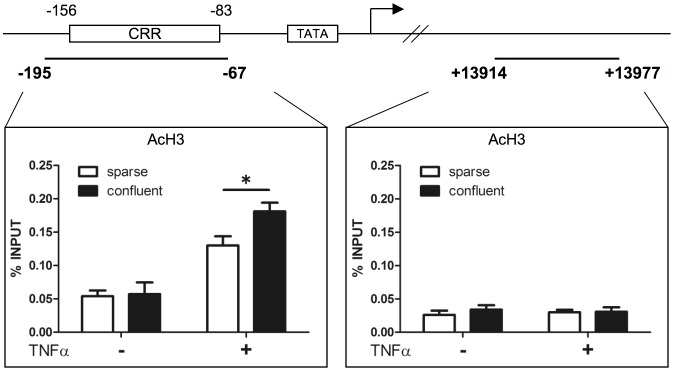
Effect of cell density on histone H3 acetylation at the E-selectin promoter. Schema of the E-selectin gene and the location of the primers used in the ChIP assay. Chromatin was prepared from sparse and confluent HUVECs monolayers treated with (+) or without (−) 1 ng/ml TNFα for 30 min, and the ChIP assay was performed using the anti-acetyl histone H3 (AcH3) antibody. Real-time PCR analysis of the ChIP samples was performed using primers designed to amplify the E-selectin promoter region (−195 to −67), which contains the cytokine response region (CRR) and the E-selectin extreme 3′ region (+13914 to +13977) (*p<0.01). The values are expressed as mean ± SD of three independent experiments.

Histone acetylation, particularly of H3, is involved in the strength, duration, and specificity of the NF-κB-activating signaling pathway [25]. As previously reported, TNFα-induced E-selectin gene expression is associated with histone acetylation [20]. Thus, we speculated that differences in histone acetylation at the E-selectin promoter may explain the difference in mRNA levels in sparse and confluent monolayers. The cytokine response region (CRR), which is located within the first 160 bp upstream of the transcriptional start site of the E-selectin gene, contains binding sites for NF-κB and activating transcription factor-2/c-JUN [23], [26], [27]. ChIP analysis of the E-selectin promoter region containing the CRR was performed using the anti-acetyl histone H3 (lysine 9 and 14) antibody. As shown in Figure 4, we determined that the histone H3 located at the E-selectin promoter was hyperacetylated after TNFα stimulation, and the acetylation level was significantly higher in the confluent monolayer than in the sparse monolayer. TNFα-induced histone acetylation was not detected at the 3′ region of the E-selectin gene, thereby indicating that histone acetylation at the E-selectin promoter is related specifically to the TNFα-induced transcription of E-selectin.

### Cell density altered the chromatin accessibility at the E-selectin promoter

**Figure 5:**
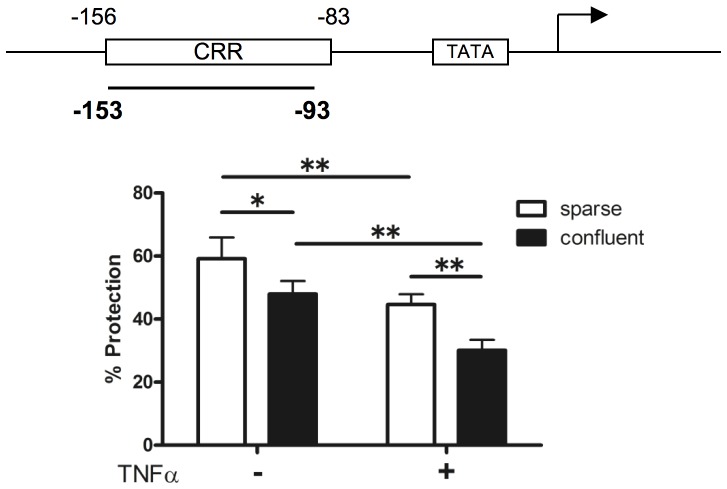
Chromatin accessibility differed between sparse and confluent HUVEC monolayers. CHART-PCR of the E-selectin promoter region. The location of primers used in CHART-PCR assays is shown. Nuclei were isolated from sparse and confluent HUVECs monolayers treated with (+) or without (−) 1 ng/ml TNFα for 60 min. The isolated nuclei were incubated with 10 U of micrococcal nuclease for 10 min at room temperature. Genomic DNA was extracted and quantified using real-time PCR relative to DNA prepared from undigested nuclei (*p<0.05, **p<0.01). The values are expressed as mean ± SD of three independent experiments. CRR indicates cytokine response region.

Histone acetylation is closely related with chromatin remodeling, which regulates gene transcription [28]; therefore, we assessed the chromatin accessibility at the E-selectin promoter region (which contains the CRR) between the two conditions. The amount of the target sequence at the E-selectin promoter region was quantified using real-time PCR after micrococcal nuclease digestion. As shown in Figure 5, the amount of the protected E-selectin target sequence was decreased significantly by TNFα stimulation, consistent with the results of a previous study [20]. We also found that the E-selectin promoter region was more accessible under the confluent condition compared with the sparse condition, even in the absence of TNFα (Figure 5).

## Discussion

In this study, we reported that TNFα-induced E-selectin expression levels were cell density dependent, and that it was regulated by the histone modification and chromatin remodeling of the E-selectin promoter. We found the following: (1) the TNFα-induced E-selectin protein and mRNA expression levels were higher in the confluent monolayer than in the sparse monolayer; (2) MAPK- and NF-κB activation were not involved in this cell density-dependent regulation; (3) TNFα-induced histone acetylation and chromatin accessibility at the E-selectin promoter region were increased in the confluent HUVEC monolayer.

Cell density apparently affects the total cellular protein contents, but we carefully excluded the effects of the cell number based on comparisons with housekeeping proteins and single cell-based flow cytometric analysis.

The difference in cell density may be affected by the extracellular matrix and the proliferation status. A previous study found that cytokine-induced nitric oxide and prostacyclin production in HUVECs were differentially regulated by the extracellular matrix [11]. We examined the effect of the extracellular matrix using fibronectin-coated dishes instead of gelatin-coated ones, but similar results were obtained (data not shown). Furthermore, we investigated the correlation between E-selectin expression levels and the cell cycle using flow cytometry, but we failed to connect specific cell cycle phase and the level of E-selectin expression between these two conditions.

Epigenetic mechanisms play an important role in regulating gene expression by altering chromatin accessibility in various tissues, including vascular endothelium [29]. Fish et al. demonstrated that EC-restricted endothelial nitric-oxide synthase expression was regulated by cell-specific histone modifications [30]. A previous study suggested an effect of TNFα stimulation on chromatin remodeling of the E-selectin promoter [20], but our data are the first to suggest that chromatin remodeling of the E-selectin promoter is altered during the maturation of the vasculature. As shown in Figure 4, the basal level of histone H3 acetylation at the E-selectin promoter was comparable between the sparse and confluent conditions. However, the induction level of histone acetylation by TNFα was decreased in the sparse monolayer. Chromatin accessibility as assessed by CHART-PCR was decreased under the sparse condition, even in the absence of TNFα. We speculate that cell density may impact the basal chromatin structure via another type of chromatin remodeling, such as another nucleosome modification or ATP-dependent chromatin remodeling. The molecular architecture responsible for cell-density-specific chromatin remodeling will be investigated in our future research.

Cell–cell interactions not only strengthens their physical structures but also affects intracellular signaling pathways [14]. Our data also suggest that the maturation of cell junctions may regulate chromatin remodeling. The cytoskeleton was mechanically connected to the lamins, a major component of nucleoskeleon, and other inner and outer nuclear membrane proteins. This complex has been indicated to regulate chromatin organization in addition to maintenance of nuclear morphology [31]. It can be hypothesized that a change in cytoskeletal organization induced by mature cell junction may impact on chromatin structure that affects gene expression.

It would appear that cell junctions are partially organized during new vessel formation when the ECs migrate and proliferate [13]. It is possible that leukocyte–EC interactions may be attenuated by decreased E-selectin expression levels during angiogenesis, which may protect vulnerable immature vessels from the inflammatory response.

In conclusion, our data are the first to demonstrate that the expression and epigenetic gene regulation of E-selectin in vascular ECs are altered by cell density. This finding suggests that the inflammatory response may change during blood vessel maturation via epigenetic mechanisms that affect the accessibility of chromatin.
